# 2946. Comparative Pharmacokinetics and Pharmacodynamics of Cefadroxil and Cephalexin for Pediatric Musculoskeletal Infections

**DOI:** 10.1093/ofid/ofad500.185

**Published:** 2023-11-27

**Authors:** Andrew Haynes, Zixuan Wei, Peter Anderson, Marc H Scheetz, Sarah K Parker, Douglas N

**Affiliations:** Children's Hospital Colorado, Aurora, CO; Skaggs School of Pharmacy and Pharmaceutical Sciences / University of Colorado Anschutz Medical Campus, Aurora, Colorado; Skaggs School of Pharmacy and Pharmaceutical Sciences / University of Colorado Anschutz Medical Campus, Aurora, Colorado; Midwestern University, Downers Grove, Illinois; University of Colorado/Children's Hospital Colorado, Aurora, CO; CU Anschutz Skaggs School of Pharmacy and Pharmaceutical Sciences, Aurora, Colorado

## Abstract

**Background:**

Methicillin-susceptible *Staphylococcus aureus* (MSSA) is one of the leading causes of pediatric musculoskeletal (MSK) infections, such as osteomyelitis and septic arthritis. MSSA MSK infections are treated initially with an IV antibiotic, followed by prolonged oral therapy. Cephalexin is the most used oral option due to long-standing clinical experience and its well-described pharmacokinetics (PK) in children. Cefadroxil could be an attractive alternative requiring less frequent dosing, but it has minimal pediatric clinical or PK data. In this study, we describe the comparative PK of cephalexin and cefadroxil in children with MSK infections and evaluate dosing regimens to achieve effective free time above the MIC (fT >MIC) for MSSA.

**Methods:**

Children aged 6 months to 18 years admitted with an MSK infection were enrolled in a prospective, open-label, crossover study and given single oral doses of cefadroxil (50-75 mg/kg up to 2,000 mg) and cephalexin (50 mg/kg up to 1,375 mg). During a single dosing interval, 4-5 plasma samples were obtained per patient for each drug. Antibiotic concentrations were quantified using liquid chromatography-mass spectrometry. PK models were developed, and then Monte Carlo simulations were performed using various dosing regimens to estimate the probability of target attainment (PTA) for a range of pharmacodynamic (PD) targets, across various pediatric ages and common MSSA MICs (1-4 mg/L).

**Results:**

Concentration-time data for cephalexin (n=15) and cefadroxil (n=14) were best described using a one-compartment model with first order absorption. PK parameters were notable for cefadroxil’s longer half-life than cephalexin. Monte Carlo simulation showed that cephalexin 120 mg/kg/day divided q8hrs (max 1,000 mg per dose) and cefadroxil 100 mg/kg/day divided q12hrs (max 1,500 mg) achieved >95% PTA for fT >MIC >40% for MICs up to 4 mg/L, though cephalexin QID and cefadroxil TID dosing were required to achieve higher PD goals.

**Conclusion:**

Among pediatric patients with MSK infections, oral cefadroxil and cephalexin both achieve PD targets for efficacy against MSSA. Twice-daily cefadroxil should be further considered as an alternative to cephalexin for oral step-down therapy given its similar PTA and possibility for less frequent dosing.

**Disclosures:**

**Marc H. Scheetz, PharmD, MSc**, Abbvie: Advisor/Consultant|ASHP: Honoraria|Chambless, Higdon, Richardson, Katz & Griggs, LLP: Expert Testimony|Cidara: Advisor/Consultant|Entasis: Advisor/Consultant|F2G: Advisor/Consultant|GSK: Advisor/Consultant|Guidepoint Global: Honoraria|Hall, Booth, Smith, P.C.: Expert Testimony|Merck: Advisor/Consultant|Reminger Co., L.P.A: Expert Testimony|Spero: Advisor/Consultant|Takeda: Advisor/Consultant|Taylor, English, Duma, LLP: Expert Testimony|Third Pole Therapeutics: Advisor/Consultant **Sarah K. Parker, MD**, Pfizer: Grant/Research Support

